# A disproportionality analysis of low molecular weight heparin in the overall population and in pregnancy women using the FDA adverse event reporting system (FAERS) database

**DOI:** 10.3389/fphar.2024.1442002

**Published:** 2024-08-12

**Authors:** Huanying Xu, Ningning Xu, Yingju Wang, Haoxi Zou, Suzhen Wu

**Affiliations:** ^1^ Foshan Clinical Medical School of Guangzhou University of Chinese Medicine, Foshan, Guangdong, China; ^2^ TCM Gynecology Department, Foshan Fosun Chancheng Hospital, Foshan, Guangdong, China

**Keywords:** low molecular weight heparin, disproportionality analysis, adverse events, pregnancy-related reports, FAERS

## Abstract

**Background:**

Low molecular weight heparin (LMWH) is extensively utilized as an anticoagulant for the prevention and management of various thrombotic conditions. However, despite the widespread use of LMWH in clinical indications, its adverse events (AEs) have not received substantial attention, and there is a lack of systematic and comprehensive AE studies. This study aims to evaluate AE signals associated with LMWH in the overall population and in pregnancy women from the FDA Adverse Event Reporting System database.

**Methods:**

We used the Standardized MedDRA Query to identify pregnancy-related AE reports. Disproportionality analyses were employed to identify LMWH-related AE by calculating the reporting odds ratios (ROR), proportional reporting ratios (PRR), bayesian confidence propagation neural network (BCPNN), and the empirical Bayesian geometric mean (EBGM).

**Results:**

For the overall population, the significantly reported adverse signals in SOCs were pregnancy, puerperium, and perinatal conditions, vascular disorders, blood and lymphatic system disorders, and product issues. The five strongest AEs signal of LMWH-related were anti factor X antibody positive (n = 6, ROR 506.70, PRR 506.65, IC 8.31, EBGM 317.03), heparin-induced thrombocytopenia test positive (n = 19, ROR 263.10, PRR 263.02, IC 7.65, EBGM 200.79), anti factor X activity increased (n = 10, ROR 255.93, PRR 255.89, IC 7.62, EBGM 196.61), heparin-induced thrombocytopenia test (n = 14, ROR 231.85, PRR 231.80, IC 7.51, EBGM 182.09), and spontaneous heparin-induced thrombocytopenia syndrome (n = 3, ROR 230.31, PRR 230.30, IC 7.50, EBGM 181.16). For pregnancy women, the five strongest AEs signals of LMWH-related included sternal fracture (n = 3, ROR 243.44, PRR 243.35, IC 6.61, EBGM 97.94), syringe issue (n = 12, ROR 97.49, PRR 97.34, IC 5.94, EBGM 61.21), bleeding time prolonged (n = 3, ROR 97.38, PRR 97.34, IC 5.94, EBGM 61.21), spinal compression fracture (n = 10, ROR 90.24, PRR 90.13, IC 5.87, EBGM 58.30), and injection site haematoma (n = 19, ROR 79.23, PRR 79.04, IC 5.74, EBGM 53.47). Additionally, unexpected AEs associated with LMWH in pregnancy women were observed, including premature baby death, placental necrosis, abortion, antiphospholipid syndrome, systolic dysfunction, compartment syndrome, body height decreased, rubella antibody positive, and ultrasound doppler abnormal.

**Conclusion:**

This study identified unexpected AE signals of LMWH-relate in pregnancy women. Our study could provide valuable evidence for the clinical practice of LMWH, especially for identifying AEs and ensuring safe usage in pregnancy women.

## 1 Introduction

Unfractionated heparin has been recognized as a significant anticoagulant and antithrombotic agent for nearly a century, originating from Jay McLean’s discovery in 1916 (Hogwood et al., 2023). Its clinical use began in the 1930s through developments by Erik Jorpes in Sweden and Charles Best in Canada (Mulloy et al., 2016). In the 1970s, researchers began modifying unfractionated heparin to enhance its pharmacological properties, leading to the development of low molecular weight heparin (LMWH) (Johnson et al., 1976). LMWHs are derived by depolymerizing unfractionated heparin (average molecular weight 15,000–19,000 Da) into smaller molecules (average molecular weight 3,500–6,000 Da) (Wang et al., 2022). Compared to unfractionated heparin, LMWH offers advantages such as increased bioavailability, longer half-lives, reduced dosing frequency, prolonged action, and a lower incidence of adverse effects. The development of LMWH began in the 1980s, with the first clinical trials conducted during this period. Enoxaparin, with an average molecular weight of 4,500 Da, was among the earliest LMWH approved for medical use, receiving initial approval in France in 1985. Subsequently, the Food and Drug Administration (FDA) approved enoxaparin in the United States in 1993, marking the widespread adoption of LMWH across various medical indications (Thompson, 2010).

LMWH is extensively utilized in clinical settings to prevent and manage various thrombotic conditions. They are frequently prescribed for ailments including deep vein thrombosis, pulmonary embolism, acute coronary syndrome, and as prophylaxis in high-risk surgical procedures (Mulloy et al., 2016). Beyond these conventional uses, LMWH has been investigated for their therapeutic potential in diverse applications (Wang et al., 2022), such as anti-cancer treatments (Ma et al., 2020), anti-viral therapies (Vitiello and Ferrara, 2023), anti-inflammatory interventions (Litov et al., 2021; Vitiello and Ferrara, 2023), antiphospholipid syndrome (Sammaritano, 2020), and recurrent spontaneous abortion (Hamulyak et al., 2020). Nevertheless, similar to all medications, LMWHs can potentially induce adverse reactions.

The most significant complication associated with LMWH treatment is bleeding, particularly when LMWHs are used for the treatment and prophylaxis of thromboembolic disorders. Clinically, the estimated incidence of bleeding ranges from 6% to 14%, which is higher than the rates observed in most clinical trials (Mulloy et al., 2016). However, the risk of bleeding is challenging to determine due to multiple factors, including the dosage and duration of heparin treatment, patient indications, the procedures undertaken, and any comedications (Hogwood et al., 2023). In addition, heparin-induced thrombocytopenia (HIT) is a potentially life-threatening complication that can occur after exposure to heparin, with a risk of 0.2% with LMWH (Martel et al., 2005). Thrombocytopenia is a rare complication that often goes unnoticed, overlooked, and does not receive timely and effective intervention especially when it is associated with LMWHs, because of its safety, ease of administration, and low incidence rate (Sahu et al., 2020). Other risks include skin lesions (Schindewolf et al., 2018), osteoporosis (Hardcastle et al., 2019; Signorelli et al., 2019; Li et al., 2022), with some reported incidence of alopecia (Sarris et al., 2003), hyperkalemia (Thomas et al., 2008; van der Heiden et al., 2022) and elevation of liver enzymes (Hahn et al., 2015; Leo et al., 2019).

Over the past several decades, the safety and efficacy of LMWH for the prevention and treatment of thrombotic conditions have been confirmed. To date, LMWH is also used in many high-risk patients, including severe renal impairment, advanced cancer, COVID-19 critically ill, and other critically ill patients (Wang et al., 2022). With the expansion of clinical indications of LMWH, we should pay more attention to whether there are new adverse reactions or whether the incidence of adverse reactions is increased. Additionally, there is increasing use of LMWH in pregnancy-related diseases, especially in recurrent miscarriage and *in vitro* fertilization, to improve pregnancy outcomes in patients (Hamulyak et al., 2020; Jiang et al., 2021), although its efficacy remains controversial (Schleussner et al., 2015; Yang XL. et al., 2018; Quenby et al., 2023). However, the safety of LMWH in pregnancy women and the monitoring of adverse reactions has not received high attention. In addition to common adverse reactions such as vaginal bleeding, oral mucosal hemorrhage, ecchymosis, and skin reactions at the injection site, there is limited clinical data on adverse event (AE) regarding abnormal fetal development, birth defects, premature delivery, abortion, and placental dysfunction potentially associated with the use of LMWH in pregnancy women. At present, due to the limited pre-clinical data, there is a lack of systematic and comprehensive adverse drug reaction studies based on real-world and big data of LMWH-related adverse events (AEs), including those on pregnancy women. Therefore, the comprehensive collection and analysis of AEs of LMWH is crucial to provide researchers with a thorough reference, promoting improvements in LMWH drug development and a comprehensive understanding of its safety.

This study intends to analyze the real-world AE signals of LMWH using the FDA Adverse Event Reporting System (FAERS) database, with a specific focus on AE signals related to LMWH in pregnancy women. First, we analyzed the AE signals of LMWH in the overall population. Then, we examined the AE signals of LMWH in the subgroup of pregnancy women. These analyses will provide insights into the AE signals of LMWH in the overall population and the subgroup of pregnancy women. Our aim is to identify AEs, reduce the risk of these reactions, and regulate the rational clinical use of LMWH.

## 2 Materials and methods

### 2.1 Study design and data source

The study sourced AE data from the FAERS, a publicly accessible database established in 2004. FAERS aggregates reports uploaded by healthcare professionals, pharmaceutical manufacturers, patients, and others, providing a standardized and voluminous dataset updated quarterly (Zhou and Hultgren, 2020). Recognized globally, FAERS serves as a pivotal reporting system and can be downloaded free from FAERS’ official website.

**FIGURE 1:**
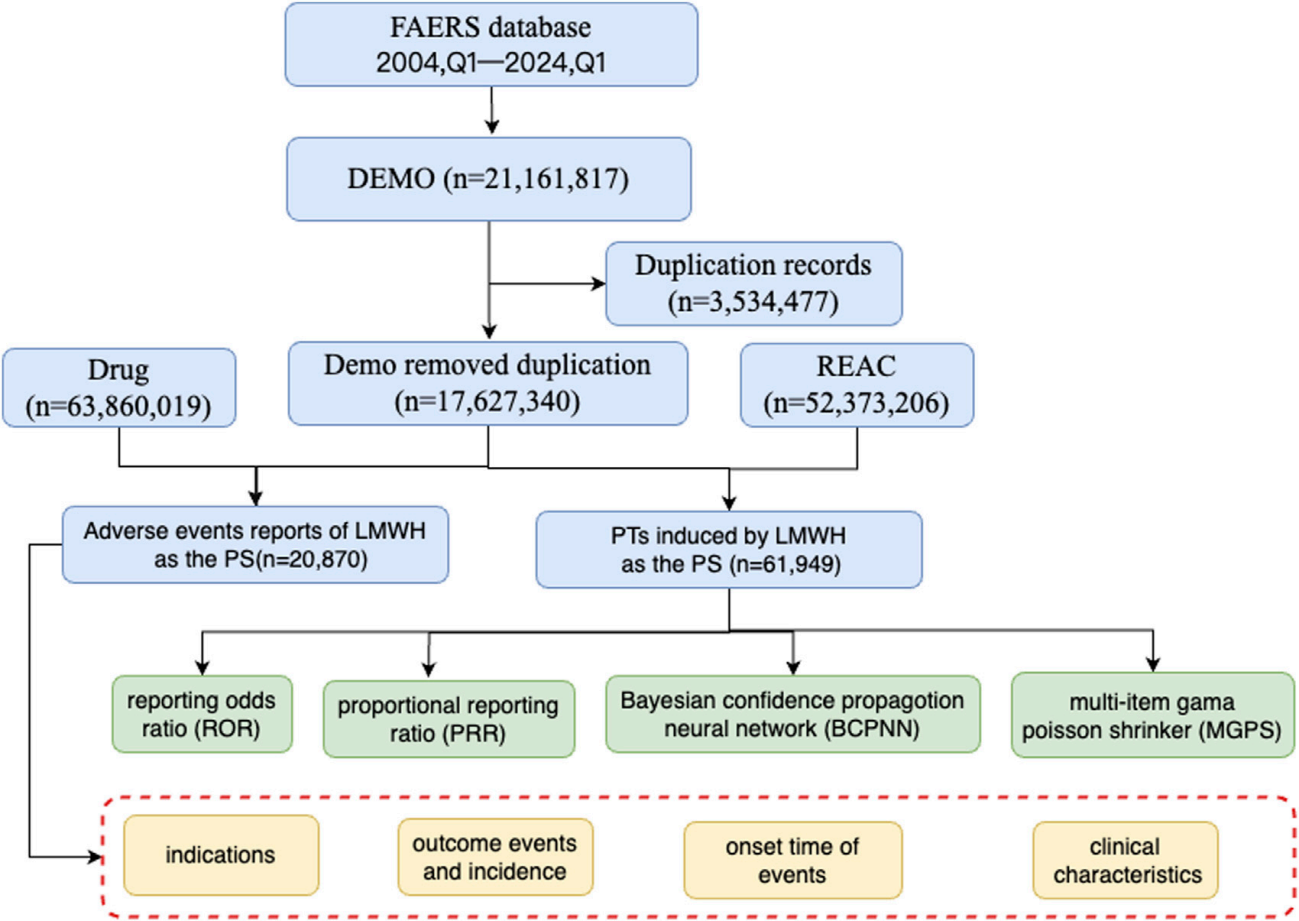
The flow diagram of selecting LMWH-related AEs in the overall population from FAERS database. Abbreviation: BCPNN, bayesian confidence propagation neural network; EBGM, empirical bayesian geometric mean; LMWH, low molecular weight heparin; MGPS, multi-item gama poisson shrinker; PTs, preferred terms; PS, primary suspect; PRR, proportional reporting ratio; ROR, reporting odds ratio.

**FIGURE 2:**
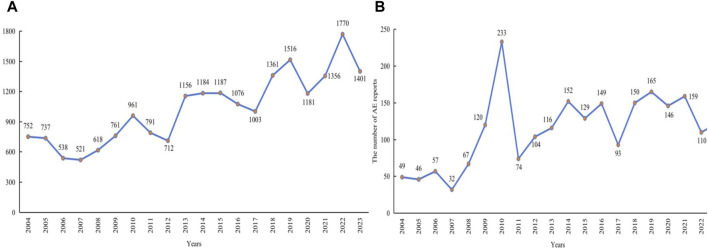
Annual distribution of LMWH-related AE reports. **(A)**, Annual distribution of LMWH-related AE reports in the overall population. **(B)**, Annual distribution of LMWH-related AE reports in pregnancy women. Abbreviation: AE, adverse event.

In this study, AE data associated with LMWH used in the overall population from 1 January 2004, to 31 March 2024, were obtained from the FAERS database, enabling a comprehensive analysis of adverse reactions related to these anticoagulants. A total of 17,627,340 AE reports were collected from FAERS, with 20,870 attributed to LMWH in the overall population. A total of 52,373,206 AEs were extracted from FAERS, of which 61,949 were associated with LMWH use in the overall population (Figure 1). The annual distribution of LMWH-related AE reports in the overall population was illustrated in Figure 2A.

### 2.2 Standardization of drug names and adverse reactions

For data with the same primary-id in the DEMO table, only the most recent report based on the date was retained. Relationships between data sets were established using the primary-id field. In addition, the Medical Dictionary for Regulatory Activities (MedDRA 27.0) was used to match the preferred terms (PTs) for LMWH adverse reactions and also listed the system organ classes (SOCs) that corresponded to these PTs.

In this study, all types of LMWH were extracted from the FAERS database, including enoxaparin, nadroparin, dalteparin, tinzaparin, bemiparin, reviparin, parnaparin, and LMWH. Data for each type of LMWH were obtained by searching for the drug name, its brand names, and product active ingredients, as listed in Supplementary Table S1. In this study, reports identifying LMWH as the primary suspect (PS) associated with AEs were extracted. These reports covered various pieces of clinical characteristics such as gender, age, reported countries, reporter, reporting time, indication, onset time of events, and outcomes of AEs.

### 2.3 Pregnancy-related report retrieval

Subgroup disproportionality analyses have been conducted due to the potential bias when analyzing the association between drugs and pregnancy outcomes in datasets in which the majority of reports are from non-pregnant women (Beyer-Westendorf et al., 2020; Sakai et al., 2022a). Since there is no dedicated field to identify reports from pregnant women in the spontaneous reporting database, efforts are being made to identify such reports using the Standardized MedDRA Query (SMQ) (Sakai et al., 2017; Sessa et al., 2019; Sakai et al., 2022a; Sakai et al., 2022b). In the current study, we utilized previously described method to identify reports of pregnancy women in the FAERS (Sakai et al., 2022a).

**FIGURE 3:**
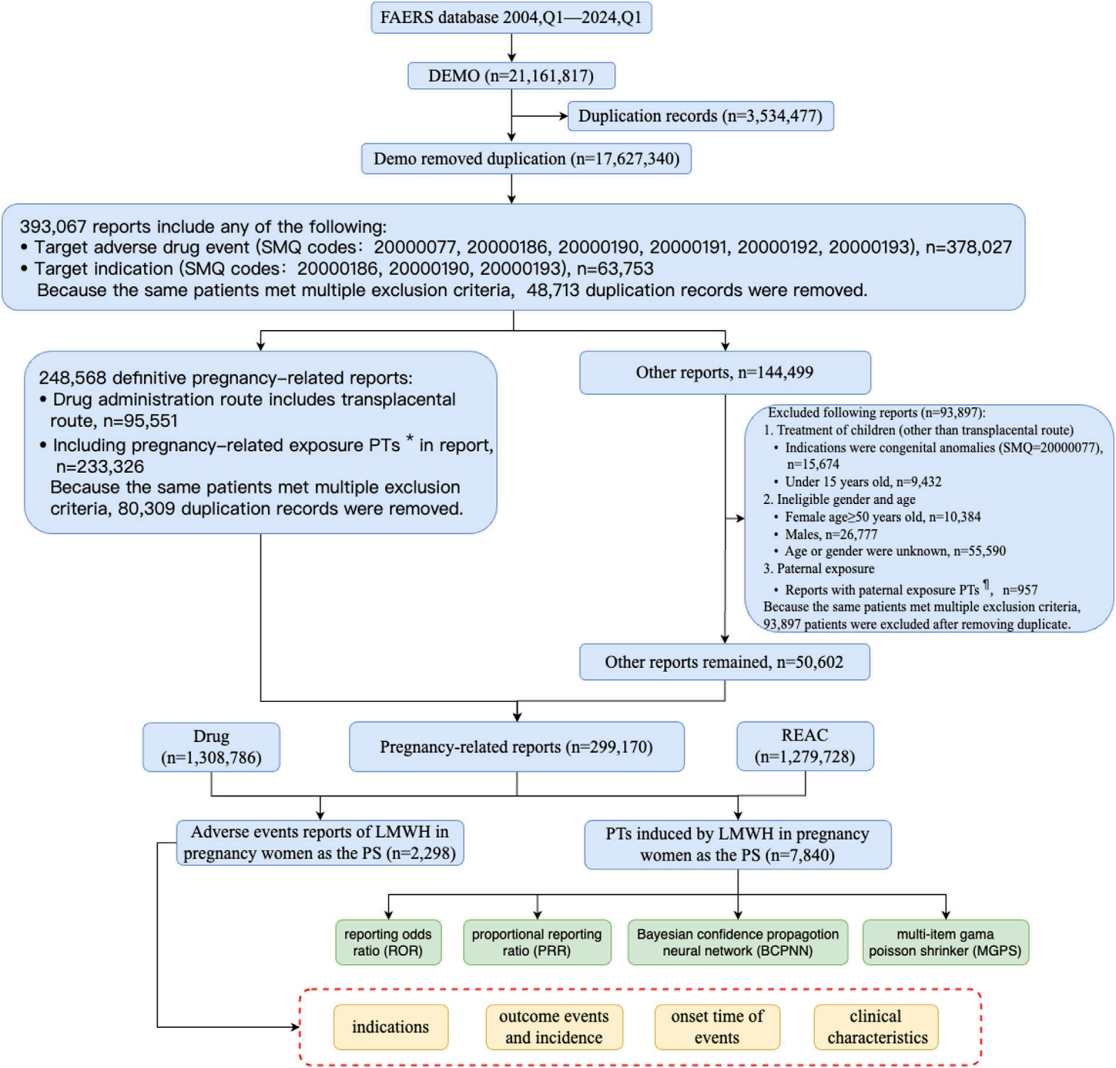
The flow diagram of selecting LMWH-related AEs in pregnancy women from FAERS database. PTs^*^: maternal exposure during delivery (10071407), foetal exposure during delivery (10071409), maternal exposure before pregnancy (10071406), maternal exposure during pregnancy (10071408), fetal exposure during pregnancy (10071404), exposure during pregnancy (10073513), maternal exposure timing unspecified (10071415), foetal exposure timing unspecified (10071405), maternal drugs affecting foetus (10026923), drug exposure before pregnancy (10064998). PTs^¶^: paternal drugs affecting the fetus (10050425), exposure via father (10071403), paternal exposure during pregnancy (10080091), paternal exposure timing unspecified (10080092), paternal exposure before pregnancy (10080093), and maternal exposure via partner during pregnancy (10084938). Abbreviation: BCPNN, bayesian confidence propagation neural network; EBGM, empirical bayesian geometric mean; LMWH, low molecular weight heparin; MGPS, multi-item gama poisson shrinker; PTs, preferred terms; PS, primary suspect; PRR, proportional reporting ratio; ROR, reporting odds ratiop; SMQ, standard MedDRA query.

Between Q1 of 2004 and Q1 of 2024, a total of 17,627,340 records were collected from FAERS, after removed duplication records (Figure 3). To obtain pregnancy-related reports, we used SMQ codes 20000077, 20000186, 20000190, 20000191, 20000192, and 20000193 in the adverse event fields, resulting in a total of 378,027 records. The breakdown of the SMQ codes were shown in Supplementary Table S2. SMQ codes 20000186, 20000190, and 20000193 were used to extract the cases that include terms related to mothers during pregnancy in the indication fields, with a total of 63,753 records. Because the same patients met multiple criteria both in the adverse event fields and in the indication fields, 393,067 patients were included after removing duplicate records.

Cases that included PTs in Supplementary Table S3 or cases in which the administration route was transplacental were defined as definitive pregnancy-related reports. After removing duplicate records, a total of 248,568 definitive pregnancy-related records remained. In addition to definitive pregnancy-related reports, 93,897 reports were excluded due to treatment of medical conditions in children, ineligible gender and age, and paternal exposure (Figure 3). After removing duplicates, 50,602 records were included for other reports. Ultimately, 299,170 reports obtained through these processes were considered pregnancy-related and were selected for our final analysis, with 2,298 attributed to LMWH in pregnancy women. A total of 1,279,728 pregnancy-related AEs were extracted, of which 7,840 were associated with LMWH in pregnancy women (shown in Figure 3). The annual distribution of LMWH-related AE reports in pregnancy women was illustrated in Figure 2B.

### 2.4 Signals analysis algorithms

In our study, we employed disproportionality analysis, a commonly utilized method in pharmacovigilance studies, to identify potential signals between drugs and AEs (Jiang et al., 2024). This widely adopted data mining approach assesses the correlation between drugs and AEs by comparing the observed frequencies in exposed and non-exposed populations through a 2 × 2 contingency table (shown in Supplementary Table S4) (Du et al., 2024). In this study, we simultaneously employed multiple methods for detecting drug adverse event signals, including the reporting odds ratios (ROR) (Rothman et al., 2004), proportional reporting ratios (PRR) (Evans et al., 2001), bayesian confidence propagation neural network (BCPNN) (Bate et al., 2002), and the empirical Bayesian geometric mean (EBGM) (Jiang et al., 2024). The calculate formula of disproportionate measurement and the criteria for signal detection are outlined in Supplementary Table S5. In our study, the four methods of ROR, PRR, BCPNN and EBGM are combined to detect signals, and the threshold is set as follows: a ≥ 3, lower limit of ROR 95% confidence interval is greater than 1, PRR ≥ 2, chi-square value ≥ 4, IC-2SD > 0, and EBGM05 > 2. The larger the values of ROR, PRR, BCPNN, and EBGM were, the stronger AE signals were, indicating a stronger statistical relationship between the target drug and the target AEs. Statistical analysis was performed using the SAS 9.4.

## 3 Results

### 3.1 Basic information about AEs of LMWH

The basic characteristics of AE reports for LMWH are summarized in Table 1. For LMWH in the overall population, female respondents contributed 48.94% of AE reports for LMWH. Most AE reports came from individuals aged 65 and older, although a significant number had unknown ages. Consumer reports were pharmacist (29.45%), and the majority of reports countries was United States (43.84%). Subcutaneous injection (46.73%) was the primary method of administration for LMWH. Serious AE outcomes for LMWH mainly consisted of other serious conditions (41.31%), hospitalization (36.42%), death (14.35%), and life-threatening situations (6.97%). The median time of AEs occurrence was 6.00 (2.00, 17.00) days for LMWH. For LMWH in pregnancy women, a significant number of reports had unknown ages (50.83%). Consumer reports primarily came from physicians (20.80%), and the majority of reports were from the United States (21.15%), although a significant number had unknown countries of report (36.03%). Subcutaneous injection (30.64%) was the primary method of administration for LMWH in pregnancy women. Serious AE outcomes for LMWH used in pregnancy mainly consisted of other serious conditions (62.84%), hospitalization (23.59%), and congenital anomalies (9.57%). The median time to AE occurrence was 15.00 (0.00, 146.00) days for LMWH in pregnancy women.

**TABLE 1:** Characteristics of AEs reports associated with LMWH.

Characteristics	LMWH used in the overall population, n (%)	LMWH used in pregnancy, n (%)
Sex		
Female	10,213 (48.94)	1,830 (79.63)
Male	7,287 (34.92)	252 (10.97)^a^
Not Specified	3,370 (16.15)	216 (9.40)
Age, years		
<18	337 (1.61)	116 (5.05)
18–44	2,534 (12.14)	993 (43.21)
45–64	3,790 (18.16)	21 (0.91)
≥65	6,928 (33.20)	NA
Not Specified	7,281 (34.89)	1,168 (50.83)
Reporter		
Consumer	5,172 (24.78)	1,024 (44.56)
Lawyer	48 (0.23)	3 (0.13)
Not Specified	1,432 (6.86)	156 (6.79)
Other health-professional	3,244 (15.54)	337 (14.66)
Pharmacist	6,146 (29.45)	300 (13.05)
Physician	4,828 (23.13)	478 (20.80)
Reported countries^b^		
United States	9,149 (43.84)	446 (19.41)
France	2,556 (12.25)	77 (3.35)
Not Specified	1,822 (8.73)	828 (36.03)
United Kingdom	964 (4.62)	48 (2.09)
Brazil	758 (3.63)	486 (21.15)
Route^b^		
Subcutaneous	9,752 (46.73)	704 (30.64)
Not Specified	5,304 (25.41)	492 (21.41)
Unknown	4,330 (20.75)	496 (21.58)
Transplacental	527 (2.53)	523 (22.76)
Parenteral	352 (1.69)	40 (1.74)
Outcomes		
Life-Threatening	1,455 (6.97)	45 (1.96)
Hospitalization - Initial or Prolonged	7,601 (36.42)	542 (23.59)
Disability	413 (1.98)	41 (1.78)
Death	2,995 (14.35)	96 (4.18)
Congenital Anomaly	237 (1.14)	220 (9.57)
Required Intervention to Prevent Permanent Impairment/Damage	354 (1.70)	13 (0.57)
Other	8,622 (41.31)	1,444 (62.84)
Adverse event occurrence time, days		
Median (Q1, Q3)	6.00 (2.00, 17.00)	15.00 (0.00, 146.00)

^a^ Gender for male was considered misreporting^.^

^b^ Only the top 5 percentages are shown.

Abbreviations: n, number of adverse events reports.

### 3.2 Signals detection associated with LMWH in the overall population

#### 3.2.1 Signals detection in SOCs levels

**FIGURE 4:**
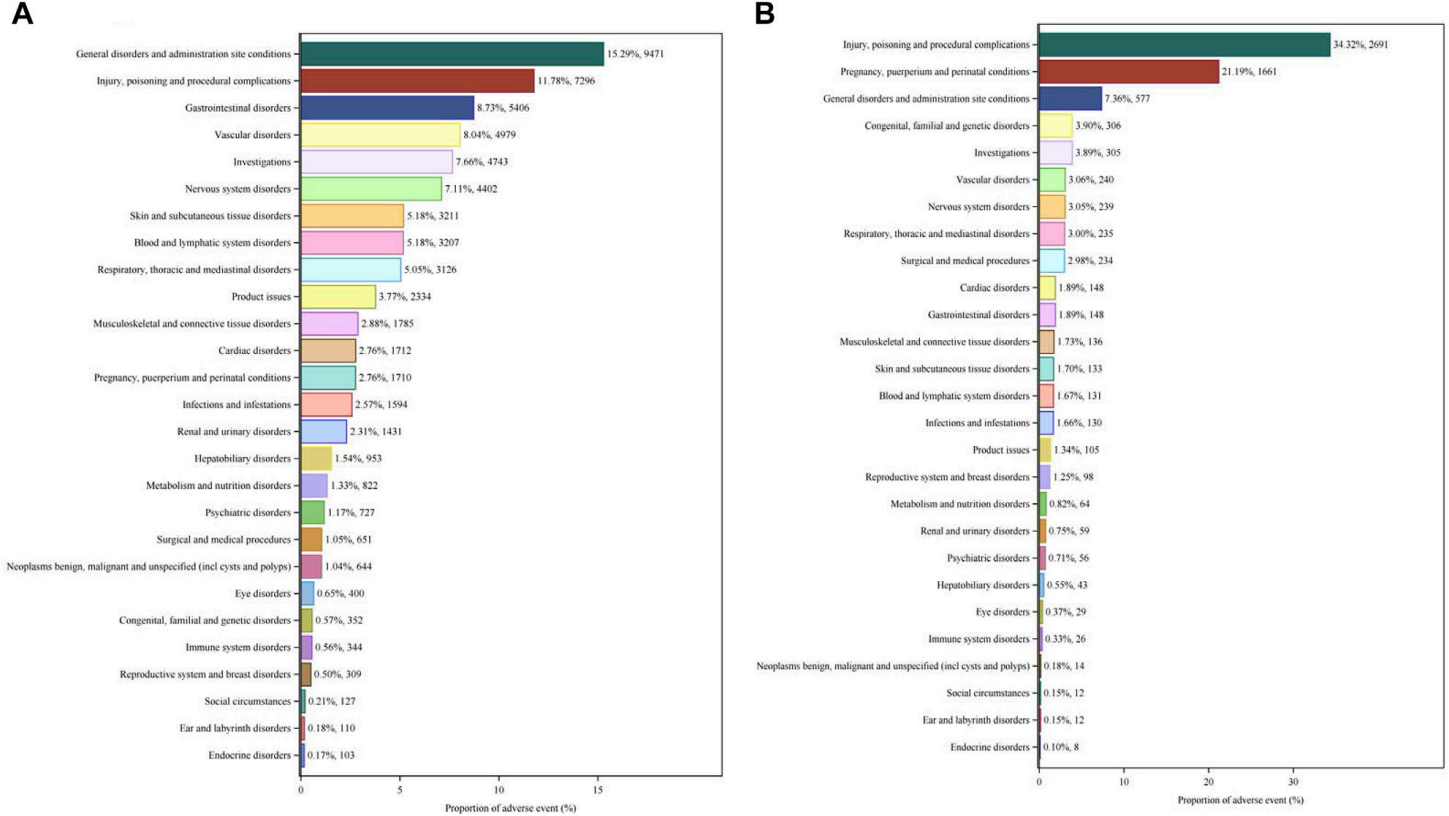
Proportion of LMWH-related AEs by SOCs. **(A)**, Proportion of LMWH-related AEs by SOCs in the overall population. **(B)**, Proportion of LMWH-related AEs by SOC in pregnancy women. The bar plot displays the statistical distribution of LMWH-related AEs across 27 SOC levels. The percentage values indicated in the figure represent the proportion of LMWH-related AEs in each SOC.

We compared the AE signals in SOCs for LWMH in the overall population, as shown in Table 2. The analysis revealed adverse reactions encompassing 27 SOCs. The proportion of LMWH-related AEs by SOCs was shown in Figure 4A. The top three proportions of LMWH-related AEs by SOCs levels were general disorders and administration site conditions (15.29%), injury, poisoning and procedural complications (11.78%), and gastrointestinal disorders (8.73%). The study findings indicated that the significantly reported adverse signals in SOCs were pregnancy, puerperium, and perinatal conditions (n = 1,710, ROR 6.54, PRR 6.39, IC 2.67, EBGM 6.34), vascular disorders (n = 4,979, ROR 3.99, PRR 3.75, IC 1.90, EBGM 3.74), blood and lymphatic system disorders (n = 3,207, ROR 3.20, PRR 3.09, IC 1.62, EBGM 3.08), and product issues (n = 2,334, ROR 2.44, PRR 2.38, IC 1.25, EBGM 2.38).

**TABLE 2:** Adverse event signals in various system organ classes for LMWH used in the overall population.

System organ class	Case reports	ROR (95% CI)	PRR (95% CI)	chi_square	IC (IC025)	EBGM (EBGM05)
Pregnancy, puerperium and perinatal conditions^a^	1710	6.54 (6.23, 6.86)	6.39 (6.09, 6.69)	7,742.02	2.67 (2.59)	6.34 (6.05)
Vascular disorders^a^	4,979	3.99 (3.88, 4.11)	3.75 (3.65, 3.85)	10,214.15	1.90 (1.86)	3.74 (3.63)
Blood and lymphatic system disorders^a^	3,207	3.20 (3.09, 3.32)	3.09 (2.99, 3.19)	4,590.15	1.62 (1.57)	3.08 (2.97)
Product issues^a^	2,334	2.44 (2.34, 2.54)	2.38 (2.29, 2.48)	1896.82	1.25 (1.19)	2.38 (2.28)
Congenital, familial and genetic disorders	352	1.86 (1.68, 2.07)	1.86 (1.67, 2.06)	139.86	0.89 (0.74)	1.86 (1.67)
Hepatobiliary disorders	953	1.69 (1.59, 1.80)	1.68 (1.58, 1.79)	265.12	0.75 (0.65)	1.68 (1.58)
Investigations	4,743	1.26 (1.22, 1.30)	1.24 (1.21, 1.28)	236.06	0.31 (0.27)	1.24 (1.20)
Renal and urinary disorders	1,431	1.21 (1.14, 1.27)	1.20 (1.14, 1.26)	48.92	0.26 (0.19)	1.20 (1.14)
Injury, poisoning and procedural complications	7,296	1.18 (1.15, 1.21)	1.16 (1.14, 1.19)	179.12	0.21 (0.18)	1.16 (1.13)
Respiratory, thoracic and mediastinal disorders	3,126	1.08 (1.04, 1.12)	1.07 (1.04, 1.11)	15.83	0.10 (0.05)	1.07 (1.03)
Cardiac disorders	1712	1.04 (0.99, 1.09)	1.04 (0.99, 1.09)	2.17	0.05 (−0.02)	1.04 (0.99)
Gastrointestinal disorders	5,406	1.03 (1.00, 1.06)	1.02 (1.00, 1.05)	3.44	0.03 (−0.01)	1.02 (1.00)
Skin and subcutaneous tissue disorders	3,211	0.96 (0.93, 1.00)	0.96 (0.93, 1.00)	4.40	−0.05 (−0.10)	0.96 (0.93)
General disorders and administration site conditions	9,471	0.85 (0.84, 0.87)	0.88 (0.86, 0.89)	199.18	−0.19 (−0.22)	0.88 (0.86)
Nervous system disorders	4,402	0.82 (0.79, 0.84)	0.83 (0.81, 0.85)	166.41	−0.27 (−0.31)	0.83 (0.81)
Surgical and medical procedures	651	0.79 (0.73, 0.85)	0.79 (0.73, 0.86)	35.90	−0.34 (−0.45)	0.79 (0.73)
Endocrine disorders	103	0.66 (0.54, 0.80)	0.66 (0.54, 0.80)	18.27	−0.60 (−0.88)	0.66 (0.54)
Metabolism and nutrition disorders	822	0.60 (0.56, 0.65)	0.61 (0.57, 0.65)	210.41	−0.71 (−0.81)	0.61 (0.57)
Reproductive system and breast disorders	309	0.55 (0.49, 0.61)	0.55 (0.49, 0.62)	113.78	−0.86 (−1.02)	0.55 (0.49)
Musculoskeletal and connective tissue disorders	1785	0.54 (0.52, 0.57)	0.56 (0.53, 0.58)	669.83	−0.85 (−0.92)	0.56 (0.53)
Immune system disorders	344	0.50 (0.45, 0.56)	0.51 (0.46, 0.56)	166.29	−0.98 (−1.13)	0.51 (0.46)
Infections and infestations	1,594	0.48 (0.46, 0.50)	0.49 (0.47, 0.52)	877.42	−1.02 (−1.09)	0.49 (0.47)
Social circumstances	127	0.44 (0.37, 0.53)	0.44 (0.37, 0.53)	89.51	−1.17 (−1.42)	0.44 (0.37)
Ear and labyrinth disorders	110	0.41 (0.34, 0.49)	0.41 (0.34, 0.49)	95.39	−1.30 (−1.56)	0.41 (0.34)
Neoplasms benign, malignant and unspecified (incl cysts and polyps)	644	0.38 (0.35, 0.41)	0.39 (0.36, 0.42)	635.81	−1.36 (−1.48)	0.39 (0.36)
Eye disorders	400	0.32 (0.29, 0.35)	0.33 (0.30, 0.36)	568.69	−1.62 (−1.76)	0.33 (0.30)
Psychiatric disorders	727	0.20 (0.18, 0.21)	0.21 (0.19, 0.22)	2,370.27	−2.28 (−2.39)	0.21 (0.19)

^a^ indicates satisfies four signal detection methods simultaneously.

Abbreviations: BCPNN, bayesian confidence propagation neural network; CI, confidence interval; EBGM, empirical bayesian geometric mean; IC, information component; PRR, proportional reporting ratio; ROR, reporting odds ratio.

#### 3.2.2 Signals detection in PTs levels

**FIGURE 5:**
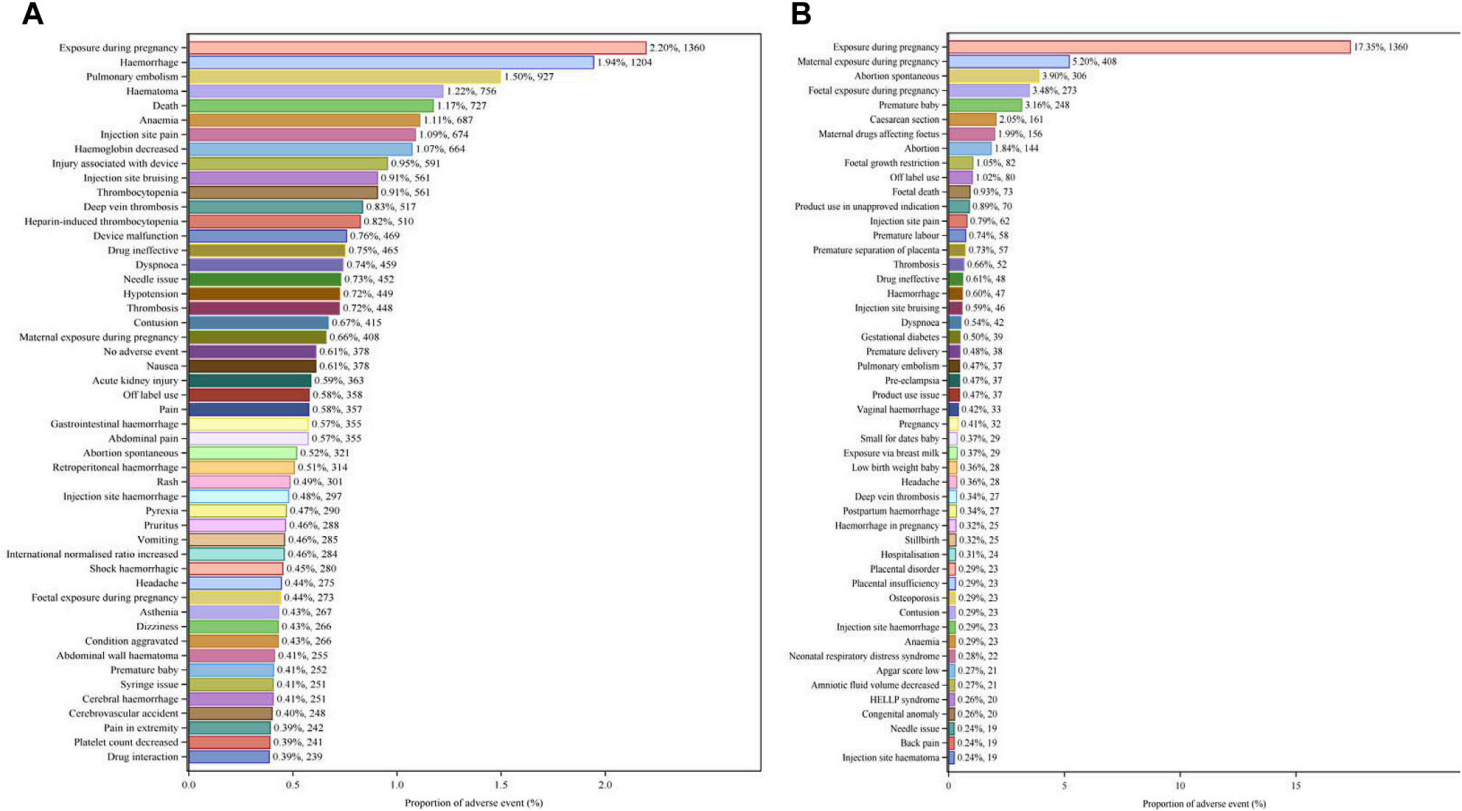
Top 50 PTs by Frequency for LMWH-related AEs. **(A)**, Top 50 PTs by frequency for LMWH-related AEs in the overall population. **(B)**, Top 50 PTs by frequency for LMWH-related AEs in pregnancy women. The bar plot displays the top 50 PTs by frequency for LMWH-related AEs. The percentages represent the number of case reports under each PT as a percentage of the total sum of all case reports.

The top 50 PTs by frequency for LMWH-related AEs in the overall population are illustrated in Figure 5A. The five PTs with the highest frequency of LMWH-related AEs were exposure during pregnancy (n = 1,360), hemorrhage (n = 1,204), pulmonary embolism (n = 927), hematoma (n = 756), and death (n = 727). Ranked based on ROR, the top 50 PTs for LMWH in the overall population are displayed in Table 3. The findings revealed PTs with high signal strength for LMWH in the overall population, including anti factor X antibody positive (n = 6, ROR 506.70, PRR 506.65, IC 8.31, EBGM 317.03), heparin-induced thrombocytopenia test positive (n = 19, ROR 263.10, PRR 263.02, IC 7.65, EBGM 200.79), anti factor X activity increased (n = 10, ROR 255.93, PRR 255.89, IC 7.62, EBGM 196.61), heparin-induced thrombocytopenia test (n = 14, ROR 231.85, PRR 231.80, IC 7.51, EBGM 182.09), and spontaneous heparin-induced thrombocytopenia syndrome (n = 3, ROR 230.31, PRR 230.30, IC 7.50, EBGM 181.16). Additionally, besides the side effects already mentioned in the instructions, unexpected adverse reactions such as placental necrosis, foetal vascular malperfusion, abortion, premature separation of placenta, premature baby death, nonreassuring fetal heart rate pattern, femoral nerve palsy, angiokeratoma, device safety feature issue, immobilisation prolonged, and removal of foreign body were also observed among the top 50 PTs associated with LMWH in the overall population.

**TABLE 3:** The significant identification of the top 50 PTs of LMWH used in the overall population.

SOC/PTs	Case reports	ROR (95% CI)	PRR (95% CI)	Chi_ square	IC (IC025)	EBGM (EBGM05)
Investigations						
Anti factor X antibody positive	6	506.70 (184.15, 1,394.23)	506.65 (184.14, 1,394.01)	1892.46	8.31 (1.48)	317.03 (115.22)
Heparin-induced thrombocytopenia test positive	19	263.10 (157.2, 440.33)	263.02 (157.17, 440.14)	3,781.55	7.65 (3.47)	200.79 (119.97)
Anti factor X activity increased	10	255.93 (126.13, 519.28)	255.89 (126.12, 519.15)	1948.46	7.62 (2.41)	196.61 (96.90)
Heparin-induced thrombocytopenia test	14	231.85 (128.34, 418.86)	231.80 (128.32, 418.72)	2,524.35	7.51 (2.97)	182.09 (100.79)
Coagulation factor XII level decreased	3	211.12 (59.57, 748.15)	211.11 (59.57, 748.08)	501.87	7.40 (0.36)	169.08 (47.71)
Factor Xa activity increased	3	168.89 (48.89, 583.42)	168.88 (48.89, 583.36)	417.23	7.14 (0.38)	140.90 (40.79)
Fibrinolysis increased	7	203.85 (89.30, 465.36)	203.83 (89.29, 465.27)	1,138.11	7.36 (1.81)	164.39 (72.01)
Coagulation factor X level increased	3	101.34 (30.59, 335.64)	101.33 (30.60, 335.61)	266.09	6.50 (0.41)	90.58 (27.35)
Ultrasound Doppler abnormal^a^	8	47.25 (23.18, 96.30)	47.24 (23.18, 96.28)	342.91	5.49 (1.94)	44.79 (21.97)
Rubella antibody positive^a^	3	46.91 (14.67, 150.05)	46.91 (14.67, 150.04)	127.71	5.48 (0.41)	44.50 (13.91)
Anti-platelet antibody positive	8	42.76 (21.02, 87.00)	42.76 (21.02, 86.99)	310.51	5.35 (1.92)	40.74 (20.02)
Injury, poisoning and procedural complications						
Exposure via blood	6	187.67 (77.48, 454.56)	187.65 (77.48, 454.48)	911.40	7.26 (1.55)	153.71 (63.46)
Foreign body in skin or subcutaneous tissue^a^	5	127.95 (49.95, 327.77)	127.94 (49.95, 327.72)	546.89	6.80 (1.25)	111.24 (43.43)
Exposure via contaminated device	3	74.51 (22.88, 242.61)	74.51 (22.88, 242.59)	199.92	6.10 (0.42)	68.55 (21.05)
Cardiac valve replacement complication	16	60.06 (36.17, 99.74)	60.05 (36.16, 99.70)	867.36	5.81 (3.00)	56.13 (33.80)
Scrotal haematoma	5	49.68 (20.16, 122.43)	49.67 (20.16, 122.41)	225.21	5.55 (1.22)	46.97 (19.06)
Extradural haematoma	62	45.61 (35.32, 58.90)	45.57 (35.29, 58.83)	2,564.10	5.44 (4.32)	43.28 (33.52)
Post procedural pulmonary embolism	10	43.09 (22.82, 81.35)	43.08 (22.82, 81.33)	391.11	5.36 (2.25)	41.04 (21.74)
Wound haematoma	6	41.53 (18.30, 94.27)	41.53 (18.30, 94.25)	226.20	5.31 (1.48)	39.63 (17.46)
Gastrointestinal disorders						
Abdominal wall haematoma	255	145.99 (127.79, 166.78)	145.39 (127.33, 166.02)	31,196.88	6.96 (6.20)	124.18 (108.71)
Retroperitoneal haemorrhage	314	133.18 (118.21, 150.04)	132.51 (117.68, 149.21)	35,424.89	6.84 (6.22)	114.67 (101.78)
Intra-abdominal haematoma	149	113.62 (95.74, 134.84)	113.35 (95.55, 134.47)	14,629.22	6.64 (5.66)	100.05 (84.31)
Retroperitoneal haematoma	210	102.08 (88.44, 117.82)	101.74 (88.19, 117.37)	18,695.35	6.51 (5.78)	90.91 (78.76)
Abdominal wall haemorrhage	21	85.28 (54.44, 133.60)	85.25 (54.43, 133.53)	1,588.24	6.28 (3.47)	77.53 (49.49)
Peritoneal haematoma	7	46.55 (21.75, 99.63)	46.54 (21.75, 99.61)	295.66	5.46 (1.74)	44.16 (20.63)
Pregnancy, puerperium and perinatal conditions						
Placental necrosis^a^	3	87.36 (26.61, 286.79)	87.35 (26.61, 286.76)	232.09	6.31 (0.42)	79.26 (24.14)
Peripartum haemorrhage	3	55.07 (17.13, 177.08)	55.07 (17.13, 177.07)	149.52	5.69 (0.42)	51.76 (16.10)
Foetal vascular malperfusion^a^	6	52.24 (22.90, 119.15)	52.23 (22.90, 119.13)	283.95	5.62 (1.51)	49.25 (21.59)
Abortion^a^	150	51.44 (43.62, 60.67)	51.32 (43.53, 60.51)	6,977.57	5.60 (4.96)	48.44 (41.07)
Premature separation of placenta^a^	59	38.45 (29.62, 49.92)	38.41 (29.59, 49.86)	2056.39	5.20 (4.14)	36.78 (28.33)
Nervous system disorders						
Spinal cord haematoma	40	141.42 (101.17, 197.68)	141.33 (101.12, 197.51)	4,774.32	6.92 (4.46)	121.21 (86.71)
Brain stem haematoma	3	64.96 (20.07, 210.21)	64.96 (20.07, 210.19)	175.42	5.92 (0.42)	60.39 (18.66)
Spinal epidural haemorrhage	4	53.62 (19.52, 147.31)	53.61 (19.52, 147.29)	194.20	5.66 (0.87)	50.47 (18.37)
Femoral nerve palsy^a^	3	47.8 (14.94, 152.97)	47.8 (14.94, 152.95)	130.09	5.50 (0.41)	45.29 (14.15)
Blood and lymphatic system disorders						
Spontaneous heparin-induced thrombocytopenia syndrome	3	230.31 (64.25, 825.57)	230.30 (64.25, 825.49)	538.14	7.50 (0.35)	181.16 (50.54)
Autoimmune heparin-induced thrombocytopenia	6	211.13 (86.30, 516.52)	211.11 (86.30, 516.43)	1,003.73	7.40 (1.55)	169.08 (69.11)
Heparin-induced thrombocytopenia	510	112.78 (102.79, 123.73)	111.86 (102.02, 122.64)	49,482.42	6.63 (6.24)	98.89 (90.14)
Musculoskeletal and connective tissue disorders						
Haematoma muscle	87	95.54 (76.53, 119.27)	95.41 (76.45, 119.07)	7,302.54	6.42 (5.13)	85.82 (68.75)
Chest wall haematoma	22	91.55 (58.96, 142.15)	91.51 (58.95, 142.08)	1776.98	6.37 (3.55)	82.66 (53.24)
Muscle haemorrhage	200	53.08 (46.00, 61.24)	52.91 (45.87, 61.02)	9,585.60	5.64 (5.12)	49.85 (43.20)
Skin and subcutaneous tissue disorders						
Bullous haemorrhagic dermatosis	21	221.74 (137.12, 358.57)	221.66 (137.09, 358.4)	3,653.86	7.46 (3.62)	175.78 (108.70)
Angiokeratoma^a^	5	62.09 (25.04, 153.99)	62.09 (25.04, 153.97)	279.95	5.86 (1.24)	57.91 (23.35)
Reproductive system and breast disorders						
Pelvic haematoma	43	106.24 (77.36, 145.91)	106.17 (77.32, 145.78)	3,979.43	6.56 (4.46)	94.42 (68.76)
Uterine haematoma	6	50.67 (22.23, 115.49)	50.67 (22.23, 115.47)	275.58	5.58 (1.51)	47.85 (21.00)
Product issues						
Device safety feature issue^a^	83	208.25 (163.77, 264.82)	207.97 (163.59, 264.40)	13,717.81	7.38 (5.46)	167.07 (131.38)
Social circumstances						
Immobilisation prolonged^a^	8	72.65 (35.28, 149.58)	72.64 (35.28, 149.55)	520.45	6.07 (2.00)	66.96 (32.52)
Endocrine disorders						
Adrenal haematoma	4	68.94 (24.88, 191.03)	68.93 (24.88, 191.01)	247.58	6.00 (0.88)	63.81 (23.03)
Surgical and medical procedures						
Removal of foreign body^a^	5	54.84 (22.19, 135.50)	54.83 (22.19, 135.48)	248.14	5.69 (1.23)	51.55 (20.86)
General disorders and administration site conditions						
Premature baby death^a^	4	49.68 (18.12, 136.18)	49.67 (18.12, 136.16)	180.17	5.55 (0.87)	46.97 (17.13)
Cardiac disorders						
Nonreassuring foetal heart rate pattern^a^	9	45.24 (23.14, 88.47)	45.24 (23.14, 88.45)	369.54	5.43 (2.11)	42.99 (21.98)

^a^ indicates unexpected AE, not mentioned in the instructions.

Abbreviations: BCPNN, bayesian confidence propagation neural network; CI, confidence interval; EBGM, empirical bayesian geometric mean; IC, information component; PRR, proportional reporting ratio; ROR, reporting odds ratio.

### 3.3 Signals detection associated with LMWH in pregnancy women

#### 3.3.1 Signals detection in SOCs levels

We compared the AE signals in SOCs for LWMH in pregnancy women, shown in Table 4. The occurrence of LMWH-related AEs among the pregnancy women encompassed 27 SOCs. Among pregnancy women, the top three proportions of LMWH-related AEs by SOCs level were injury, poisoning, and procedural complications (34.32%), pregnancy, puerperium, and perinatal conditions (21.19%), and general disorders and administration site conditions (7.36%), shown in Figure 4B. The significantly reported adverse signals in SOCs were product issues (n = 105, ROR 2.99, PRR 2.97, IC 1.55, EBGM 2.93), vascular disorders (n = 240, ROR 2.79, PRR 2.74, IC 1.44, EBGM 2.71), and blood and lymphatic system disorders (n = 131, ROR 2.07, PRR 2.05, IC 1.03, EBGM 2.04).

**TABLE 4:** Adverse event signals in various system organ classes for LMWH in pregnancy women.

System organ class	Case reports	ROR (95% CI)	PRR (95% CI)	chi_square	IC (IC025)	EBGM (EBGM05)
Product issues^a^	105	2.99 (2.46, 3.63)	2.97 (2.45, 3.59)	134.91	1.55 (1.24)	2.93 (2.41)
Vascular disorders^a^	240	2.79 (2.45, 3.18)	2.74 (2.41, 3.10)	263.10	1.44 (1.24)	2.71 (2.38)
Blood and lymphatic system disorders^a^	131	2.07 (1.74, 2.46)	2.05 (1.73, 2.43)	70.26	1.03 (0.76)	2.04 (1.71)
Injury, poisoning and procedural complications	2,691	1.63 (1.56, 1.71)	1.41 (1.37, 1.46)	428.14	0.50 (0.43)	1.41 (1.35)
Surgical and medical procedures	234	1.52 (1.34, 1.74)	1.51 (1.33, 1.71)	40.41	0.59 (0.39)	1.50 (1.32)
Pregnancy, puerperium and perinatal conditions	1,661	1.51 (1.43, 1.59)	1.40 (1.34, 1.46)	221.02	0.48 (0.40)	1.40 (1.32)
Investigations	305	1.05 (0.94, 1.18)	1.05 (0.94, 1.17)	0.72	0.07 (−0.10)	1.05 (0.93)
Renal and urinary disorders	59	1.01 (0.78, 1.31)	1.01 (0.79, 1.31)	0.01	0.02 (−0.36)	1.01 (0.78)
Cardiac disorders	148	0.96 (0.82, 1.14)	0.96 (0.82, 1.13)	0.19	−0.05 (−0.29)	0.97 (0.82)
Respiratory, thoracic and mediastinal disorders	235	0.95 (0.84, 1.09)	0.95 (0.84, 1.08)	0.52	−0.07 (−0.26)	0.96 (0.84)
Reproductive system and breast disorders	98	0.95 (0.78, 1.16)	0.95 (0.78, 1.16)	0.26	−0.07 (−0.37)	0.95 (0.78)
Hepatobiliary disorders	43	0.93 (0.69, 1.25)	0.93 (0.69, 1.25)	0.25	−0.11 (−0.55)	0.93 (0.69)
Skin and subcutaneous tissue disorders	133	0.75 (0.63, 0.89)	0.75 (0.64, 0.89)	11.07	−0.41 (−0.66)	0.75 (0.63)
General disorders and administration site conditions	577	0.72 (0.66, 0.78)	0.74 (0.68, 0.80)	58.03	−0.43 (−0.56)	0.74 (0.68)
Nervous system disorders	239	0.68 (0.60, 0.78)	0.69 (0.61, 0.78)	34.25	−0.53 (−0.72)	0.69 (0.61)
Metabolism and nutrition disorders	64	0.64 (0.50, 0.82)	0.64 (0.50, 0.82)	12.85	−0.64 (−0.99)	0.64 (0.50)
Endocrine disorders	8	0.60 (0.30, 1.21)	0.60 (0.30, 1.21)	2.07	−0.72 (−1.62)	0.61 (0.30)
Immune system disorders	26	0.59 (0.40, 0.87)	0.59 (0.40, 0.87)	7.44	−0.76 (−1.29)	0.59 (0.40)
Infections and infestations	130	0.55 (0.46, 0.65)	0.56 (0.47, 0.66)	46.96	−0.84 (−1.09)	0.56 (0.47)
Eye disorders	29	0.51 (0.35, 0.73)	0.51 (0.35, 0.73)	13.94	−0.98 (−1.48)	0.51 (0.35)
Ear and labyrinth disorders	12	0.49 (0.28, 0.87)	0.50 (0.28, 0.87)	6.18	−1.01 (−1.76)	0.50 (0.28)
Neoplasms benign, malignant and unspecified (incl cysts and polyps)	14	0.44 (0.26, 0.75)	0.44 (0.26, 0.75)	9.71	−1.16 (−1.86)	0.45 (0.26)
Congenital, familial and genetic disorders	306	0.44 (0.40, 0.50)	0.47 (0.42, 0.52)	204.45	−1.10 (−1.26)	0.47 (0.42)
Musculoskeletal and connective tissue disorders	136	0.43 (0.37, 0.51)	0.44 (0.38, 0.52)	98.72	−1.17 (−1.41)	0.44 (0.38)
Gastrointestinal disorders	148	0.43 (0.36, 0.50)	0.44 (0.37, 0.51)	111.30	−1.19 (−1.42)	0.44 (0.37)
Social circumstances	12	0.32 (0.18, 0.56)	0.32 (0.18, 0.57)	17.23	−1.63 (−2.36)	0.32 (0.18)
Psychiatric disorders	56	0.15 (0.11, 0.19)	0.15 (0.12, 0.20)	274.65	−2.70 (−3.06)	0.15 (0.12)

^a^ indicates satisfies four signal detection methods simultaneously.

Abbreviations: BCPNN, bayesian confidence propagation neural network; CI, confidence interval; EBGM, empirical bayesian geometric mean; IC, information component; PRR, proportional reporting ratio; ROR, reporting odds ratio.

#### 3.3.2 Signals detection in PTs levels

The five PTs with the highest frequency of LMWH-related AEs were exposure during pregnancy (n = 1,360), maternal exposure during pregnancy (n = 408), abortion spontaneous (n = 306), foetal exposure during pregnancy (n = 273), and premature baby (n = 248), shown in Figure 5B. Ranked based on ROR, the top 50 PTs for LMWH in pregnancy women are displayed in Table 5. The five strongest signals for LMWH-related among the pregnancy women included sternal fracture (n = 3, ROR 243.44, PRR 243.35, IC 6.61, EBGM 97.94), syringe issue (n = 12, ROR 97.49, PRR 97.34, IC 5.94, EBGM 61.21), bleeding time prolonged (n = 3, ROR 97.38, PRR 97.34, IC 5.94, EBGM 61.21), spinal compression fracture (n = 10, ROR 90.24, PRR 90.13, IC 5.87, EBGM 58.30), and injection site haematoma (n = 19, ROR 79.23, PRR 79.04, IC 5.74, EBGM 53.47). Additionally, unexpected AEs associated with LMWH in pregnancy women were observed, including premature baby death, placental necrosis, abortion, antiphospholipid syndrome, systolic dysfunction, compartment syndrome, body height decreased, rubella antibody positive, and ultrasound doppler abnormal.

**TABLE 5:** The significant identification of the top 50 PTs of LMWH in pregnancy women.

SOC/PTs	Case reports	ROR (95% CI)	PRR (95% CI)	chi_square	IC (IC025)	EBGM (EBGM05)
Injury, poisoning and procedural complications						
Sternal fracture	3	243.44 (40.67, 1,457.14)	243.35 (40.67, 1,456.18)	289.62	6.61 (0.09)	97.94 (16.36)
Spinal compression fracture	10	90.24 (41.64, 195.56)	90.13 (41.62, 195.18)	566.62	5.87 (2.20)	58.30 (26.90)
Cardiac valve replacement complication	11	59.57 (29.84, 118.9)	59.48 (29.82, 118.66)	462.83	5.45 (2.31)	43.79 (21.94)
Spinal fracture	11	55.84 (28.14, 110.83)	55.77 (28.12, 110.59)	440.30	5.38 (2.30)	41.76 (21.04)
Femur fracture	5	21.36 (8.4, 54.28)	21.35 (8.40, 54.22)	85.69	4.25 (0.98)	18.98 (7.47)
Incorrect dose administered by device	3	20.29 (6.11, 67.38)	20.28 (6.11, 67.33)	48.88	4.18 (0.23)	18.14 (5.46)
Recalled product administered	3	18.73 (5.67, 61.88)	18.72 (5.67, 61.83)	45.11	4.08 (0.22)	16.89 (5.11)
Product storage error	13	13.81 (7.83, 24.33)	13.78 (7.83, 24.27)	142.09	3.68 (1.99)	12.78 (7.25)
General disorders and administration site conditions						
Injection site haematoma	19	79.23 (45.76, 137.15)	79.04 (45.7, 136.7)	984.40	5.74 (3.13)	53.47 (30.89)
Injury associated with device	7	29.14 (13.03, 65.18)	29.12 (13.03, 65.08)	161.15	4.63 (1.53)	24.84 (11.11)
Injection site haemorrhage	23	24.62 (15.87, 38.19)	24.55 (15.84, 38.04)	451.32	4.42 (2.90)	21.45 (13.83)
Injection site bruising	46	19.70 (14.49, 26.77)	19.59 (14.44, 26.58)	724.18	4.14 (3.25)	17.58 (12.94)
Injection site discolouration	8	15.84 (7.66, 32.75)	15.83 (7.66, 32.70)	101.25	3.86 (1.52)	14.51 (7.02)
Application site pain	3	14.32 (4.40, 46.63)	14.31 (4.40, 46.6)	34.14	3.73 (0.18)	13.23 (4.06)
Premature baby death^a^	4	10.64 (3.87, 29.28)	10.64 (3.87, 29.25)	32.78	3.33 (0.49)	10.04 (3.65)
Vascular disorders						
Peripheral ischaemia	4	59.02 (18.79, 185.4)	58.99 (18.79, 185.23)	167.23	5.44 (0.71)	43.53 (13.86)
Embolism	9	52.20 (24.63, 110.67)	52.15 (24.61, 110.47)	341.67	5.31 (1.99)	39.70 (18.73)
Pelvic venous thrombosis	3	17.39 (5.29, 57.21)	17.38 (5.29, 57.16)	41.84	3.98 (0.21)	15.80 (4.80)
Thrombosis	52	14.19 (10.68, 18.86)	14.11 (10.64, 18.71)	582.89	3.71 (3.00)	13.06 (9.83)
Haematoma	18	13.30 (8.22, 21.52)	13.27 (8.21, 21.45)	188.86	3.63 (2.26)	12.35 (7.63)
Ischaemia	3	9.55 (2.98, 30.59)	9.54 (2.98, 30.57)	21.67	3.18 (0.09)	9.07 (2.83)
Investigations						
Bleeding time prolonged	3	97.38 (23.27, 407.53)	97.34 (23.27, 407.24)	178.78	5.94 (0.19)	61.21 (14.63)
Body height decreased^a^	3	54.10 (14.64, 199.86)	54.08 (14.64, 199.71)	117.22	5.35 (0.24)	40.81 (11.05)
Rubella antibody positive^a^	3	23.18 (6.91, 77.74)	23.18 (6.91, 77.68)	55.70	4.35 (0.24)	20.40 (6.08)
Ultrasound Doppler abnormal^a^	7	21.05 (9.57, 46.27)	21.03 (9.57, 46.20)	118.22	4.23 (1.45)	18.73 (8.52)
Fibrin D dimer increased	6	14.32 (6.21, 33.02)	14.31 (6.21, 32.97)	68.29	3.73 (1.12)	13.23 (5.74)
Product issues						
Syringe issue	12	97.49 (47.64, 199.49)	97.34 (47.60, 199.05)	715.13	5.94 (2.49)	61.21 (29.91)
Needle issue	19	65.74 (38.57, 112.06)	65.58 (38.51, 111.69)	860.54	5.55 (3.09)	46.99 (27.57)
Product packaging issue	7	51.66 (22.06, 120.98)	51.62 (22.06, 120.80)	263.60	5.30 (1.61)	39.40 (16.83)
Product availability issue	11	14.53 (7.84, 26.93)	14.51 (7.83, 26.87)	127.01	3.74 (1.85)	13.40 (7.23)
Device defective	3	11.87 (3.68, 38.36)	11.87 (3.68, 38.33)	27.83	3.48 (0.14)	11.13 (3.45)
Musculoskeletal and connective tissue disorders						
Osteoporotic fracture	5	73.79 (25.63, 212.42)	73.74 (25.63, 212.19)	246.66	5.67 (1.07)	51.01 (17.72)
Compartment syndrome^a^	3	40.57 (11.45, 143.81)	40.56 (11.45, 143.70)	92.60	5.03 (0.25)	32.65 (9.21)
Osteoporosis	23	14.28 (9.32, 21.88)	14.24 (9.3, 21.80)	260.38	3.72 (2.51)	13.17 (8.60)
Osteopenia	8	9.28 (4.55, 18.93)	9.27 (4.55, 18.90)	55.84	3.14 (1.24)	8.82 (4.33)
Pregnancy, puerperium and perinatal conditions						
Placental necrosis^a^	3	17.39 (5.29, 57.21)	17.38 (5.29, 57.16)	41.84	3.98 (0.21)	15.80 (4.80)
Peripartum haemorrhage	3	12.17 (3.76, 39.35)	12.17 (3.76, 39.32)	28.60	3.51 (0.15)	11.39 (3.52)
Abortion^a^	144	11.36 (9.58, 13.47)	11.17 (9.45, 13.20)	1,249.27	3.39 (3.05)	10.51 (8.87)
Blood and lymphatic system disorders						
Hypercoagulation	14	22.53 (12.87, 39.41)	22.49 (12.86, 39.31)	252.48	4.31 (2.34)	19.87 (11.36)
Antiphospholipid syndrome^a^	10	17.85 (9.29, 34.31)	17.83 (9.28, 34.24)	143.12	4.01 (1.84)	16.16 (8.41)
Heparin-induced thrombocytopenia	4	14.76 (5.3, 41.08)	14.75 (5.30, 41.04)	46.99	3.77 (0.59)	13.60 (4.89)
Nervous system disorders						
Cerebral thrombosis	4	18.55 (6.59, 52.2)	18.54 (6.59, 52.15)	59.57	4.07 (0.64)	16.74 (5.95)
Ischaemic stroke	5	11.12 (4.49, 27.52)	11.11 (4.49, 27.49)	43.06	3.39 (0.79)	10.46 (4.23)
Transient ischaemic attack	7	9.63 (4.49, 20.65)	9.62 (4.49, 20.62)	51.07	3.19 (1.12)	9.14 (4.26)
Cardiac disorders						
Cardiac ventricular thrombosis	11	36.47 (18.96, 70.16)	36.42 (18.94, 70.01)	309.46	4.90 (2.22)	29.93 (15.56)
Systolic dysfunction^a^	3	11.07 (3.44, 35.64)	11.06 (3.44, 35.62)	25.70	3.38 (0.13)	10.42 (3.23)
Skin and subcutaneous tissue disorders						
Livedo reticularis	3	9.55 (2.98, 30.59)	9.54 (2.98, 30.57)	21.67	3.18 (0.09)	9.07 (2.83)
Respiratory, thoracic and mediastinal disorders						
Pulmonary thrombosis	4	19.67 (6.97, 55.55)	19.66 (6.97, 55.49)	63.20	4.14 (0.65)	17.65 (6.25)
Reproductive system and breast disorders						
Uterine haematoma	5	12.30 (4.95, 30.53)	12.29 (4.95, 30.50)	48.21	3.52 (0.83)	11.50 (4.63)
Gastrointestinal disorders						
Abdominal wall haematoma	3	24.34 (7.23, 81.94)	24.33 (7.23, 81.88)	58.37	4.41 (0.24)	21.29 (6.33)

^a^ indicates unexpected AE, not mentioned in the instructions.

Abbreviations: BCPNN, bayesian confidence propagation neural network; CI, confidence interval; EBGM, empirical bayesian geometric mean; IC, information component; PRR, proportional reporting ratio; ROR, reporting odds ratio.

## 4 Discussion

Our pharmacovigilance analysis of the FAERS database comprehensively and systematically revealed the safety signals of LMWH. For the overall population, significant AEs at SOC levels included pregnancy, puerperium, and perinatal conditions; vascular disorders; blood and lymphatic system disorders; and product issues. The five strongest AE signals were anti factor X antibody positive, heparin-induced thrombocytopenia test positive, anti factor X activity increased, heparin-induced thrombocytopenia test, and spontaneous heparin-induced thrombocytopenia syndrome. Besides the side effects of bleeding, hematoma, and HIT mentioned in the drug’s instructions, unexpected AEs such as placental necrosis, foetal vascular malperfusion, abortion, premature separation of placenta, premature baby death, nonreassuring fetal heart rate pattern, femoral nerve palsy, angiokeratoma, device safety feature issue, immobilisation prolonged, and removal of foreign body were also observed. Additionally, for pregnancy women, significant AEs at SOC levels included product issues, vascular disorders, and blood and lymphatic system disorders. The five strongest AE signals were sternal fracture, syringe issue, prolonged bleeding time, spinal compression fracture, and injection site hematoma. Unexpected AEs associated with LMWH in pregnancy women were observed, including premature baby death, placental necrosis, abortion, antiphospholipid syndrome, systolic dysfunction, compartment syndrome, decreased body height, positive rubella antibody, and abnormal ultrasound doppler results.

The most important complication associated with heparin treatment is bleeding, resulting directly from the potency of heparin as an anticoagulant. As we know, all antithrombotic carry the risk of bleeding as a complication (Schulman et al., 2008). The incidence of bleeding under heparin therapy is hard to define, as it depends on numerous parameters including the indication, dosage, duration of heparin application, baseline status of the patient, procedure that the patient undergoes, and co-medication (Alban, 2012). In all patients treated with antithrombotic therapy, increased age, and renal damage are strong independent baseline predictors of major bleeding (Lim et al., 2006; Clark, 2008; Eikelboom et al., 2009). In general, bleeding can occur at any site during therapy. Major bleeds are rare adverse events, but hematomas are commonly observed (Alban, 2012). An estimate of the incidence of bleeding clinically is between 6% and 14% (Mulloy et al., 2016).

HIT is a life-threatening severe nonbleeding adverse reaction caused by LMWH (Dhakal et al., 2018). Nonbleeding complications of heparins are induced by binding of heparin molecules to proteins other than antithrombin and to cells, which is generally more pronounced with unfractionated heparin (with an incidence of about 2.5%) than with LMWH(with an incidence of about 0.2%) (Hogwood et al., 2023). HIT can be divided into type I and type II. Type I is an early-onset, mild thrombocytopenia that does not lead to thromboembolism, whereas type II is immune-mediated and clinically severe, causing both thrombocytopenia and thromboembolism (Arepally and Padmanabhan, 2021), with approximately 50% of patients developing arterial or venous thrombosis usually occurring 5–10 days after starting heparin therapy (Barcellona et al., 2020; Singh et al., 2020; Liu et al., 2023). The immunologic nature of the more serious type II HIT is due to the generation of antibodies that recognize complexes of heparin and PLT factor 4. PLT factor 4 is a positively charged protein released from the α-granules of activated platelets and combines with the negatively charged heparin through electrostatic interaction of the vascular epidermis to form a complex (Hogwood et al., 2023; Mongirdiene et al., 2023), which results in thrombocytopenia and increases the risk of venous and arterial thromboses (Arepally, 2017; Rollin et al., 2022). In this study, we found HIT was the strongest signal, followed by spontaneous haematoma and autoimmune heparin−induced thrombocytopenia in blood and lymphatic system disorders. Ranked based on ROR, the top 6 PTs for LMWH were anti factor x antibody positive, heparin-induced thrombocytopenia test positive, anti factor x activity increased, heparin-induced thrombocytopenia test, factor xa activity increased, and spontaneous heparin-induced thrombocytopenia. Our findings were consistent with previous reports (Liu et al., 2023).

LMWHs are increasingly used during pregnancy, either as prophylaxis for venous thromboembolism or as an effective treatment of acute venous thromboembolism in pregnancy (Bates et al., 2018; Bistervels et al., 2022). Other clinically practiced indications for LMWH use in pregnancy-related diseases include unexplained recurrent miscarriage, thrombophilia, autoimmune disease, and *in vitro* fertilization to improve pregnancy rates and outcomes. In addition, early studies suggested an association between placenta‐mediated complications in pregnancy and women with thrombophilia or an increased risk of developing thrombosis (Rodger et al., 2010). As a result, physicians have prescribed heparin as prophylaxis to prevent placenta-mediated pregnancy complications (Duffett and Rodger, 2015; Cruz-Lemini et al., 2022; Zullino et al., 2022). However, the evidence that LMWH can reduce the risk of placenta-mediated pregnancy complications including late pregnancy loss, placental abruption, pre-eclampsia, small-for-gestational-age neonate, and fetal growth restriction, is controversial (Mastrolia et al., 2016; Rodger et al., 2016). Although there is no evidence that heparins cross the placenta, and consequently no fetal or neonatal complication has been reported (Duffett and Rodger, 2015), the safety issues should cause our concern, especially regarding abnormal fetal development, birth defects, premature delivery, abortion, and placental dysfunction.

In our study, besides side effects of hematoma and bruising or haemorrhage at the injection site, unexpected AEs about fetal induced by LMWH in pregnancy women were observed, including premature baby death, placental necrosis, abortion, and abnormal ultrasound doppler results. Previous reports indicated that treatment with a prophylactic dose of LMWH during pregnancy was related to an increased risk of prematurity complications (Isma et al., 2010). While fetal risk was low in LMWH exposed pregnancies (Sogaard et al., 2022), and neonatal death related to LMWH was limited, our findings should be cautiously explored. Large studies are needed to better confirm the findings. In addition, caesarean section is an important risk factor for *postpartum* venous thrombosis in pregnant women. However, due to the lack of evidence, the benefits of pharmacologic thromboprophylaxis such as LMWH in preventing the occurrence of venous thrombosis in caesarean section patients remain controversial (Yang R. et al., 2018). Results from a previous meta-analysis showed that LMWH was associated with no obvious decrease in the risk of thrombus after caesarean section compared with UHF and negative control. However, LMWH was observed to be associated with a definite increase in the risk of bleeding or hematomas after caesarean section in comparison to negative control (Yang R. et al., 2018). Similarly, our study found the AE of uterine haematoma, peripartum haemorrhage for LMWH use in pregnancy women. Contrary, recent research suggested that the use of LMWH was not associated with increased critical obstetric bleeding among women with caesarean section (Akaishi et al., 2024). Due to inconsistent findings, large studies are needed to better confirm the safety of LMWH for women undergoing caesarean section.

It is worth noting that fractures and osteoporosis are the most significant AE signals related to LMWH in pregnancy women in this study. Data on the effects of LMWH on bone loss and fractures in pregnant women are limited. Some reports have shown that at least 3 months of LMWH use was associated with bone loss and fractures in the pregnancy women, with more significant reductions observed in patients receiving enoxaparin for more than 1 year (Signorelli et al., 2019). However, an observational cohort study of 152 pregnant women found that prolonged use of LMWH during pregnancy was not related to a subsequent decrease in BMD (measured by dual-energy X-ray absorptiometry 4–7 years after the last delivery), osteopenia, osteoporosis, or osteoporotic fractures (Galambosi et al., 2016). Therefore, based on current literature findings, long-term LMWH exposure during pregnancy may have some negative effect on BMD. Large sample sizes and long-term follow-up studies are needed to better confirm the association between bone loss and LMWH in pregnant women.

Our study has several limitations. Firstly, adverse event reports are voluntary and come from a variety of sources, resulting in varying degrees of underreporting, delayed reporting, misreporting, and incomplete information, which may introduce bias into the measurement of the disproportionality report. Secondly, disproportionality analysis alone can be a useful step in identifying safety signals; however, it cannot formally establish causation or measure incidence due to limitations such as lack of detailed patient exposure data, reporting biases, and confounding factors. Thirdly, our study cannot directly explain the mechanism underlying the development of some unexpected adverse reactions caused by LMWHs, which requires specific mechanisms to be studied through cell and animal experiments in the future. Finally, the lack of multivariable analyses controlling for other clinical factors such as age, comorbidities, or other factors, makes it challenging to implicate the specific role of LMWH in the development of adverse reactions. Considering the above limitations and other potential biases, we need to interpret the results more cautiously, and further clinical study evaluations are required to confirm these associations.

## 5 Conclusion

In conclusion, the significant AEs at SOC levels related to LMWH were pregnancy, puerperium, and perinatal conditions, vascular disorders, blood and lymphatic system disorders, and product issues. Bleeding, hematoma, and HIT are common AEs for which patients should be monitored. Additionally, the five strongest AE signals for LMWH-related in pregnancy women were sternal fracture, syringe issue, prolonged bleeding time, spinal compression fracture, and injection site hematoma. Unexpected AEs associated with LMWH in pregnancy women were observed, including premature baby death, placental necrosis, abortion, antiphospholipid syndrome, systolic dysfunction, compartment syndrome, decreased body height, positive rubella antibody, and abnormal ultrasound doppler results. Our study could provide valuable evidence for the clinical practice of LMWH, especially for identifying AEs and ensuring safe usage in pregnancy women.

## Data Availability

The original contributions presented in the study are included in the article/Supplementary Material, further inquiries can be directed to the corresponding authors.
