# Supplementary material for: A disproportionality analysis of low molecular weight heparin in the overall population and in pregnancy women using the FDA adverse event reporting system (FAERS) database
Source: Front Pharmacol. 2024 Aug 12;15:1442002. doi: 10.3389/fphar.2024.1442002 (PMC11345143; doi:10.3389/fphar.2024.1442002)
Supplement: Supplementary file 1 [file Table1.DOCX]

**Supplementary Data**

Table 1S. The drug name and product active ingredient for each type of LMWH searching FAERS database

| Drug name/brand name | Product active ingredient |
| --- | --- |
| enoxaparin/ lovenox/ clexane | enoxaparin sodium |
| dalteparin/ fragmin | dalteparin sodium |
| tinzaparin/ innohep | tinzaparin sodium |
| nadroparin/ fraxiparin | nadroparin calcium |
| bemiparin/ hibor | bemiparin sodium |
| parnaparin/ fluxum | parnaparin sodium |
| reviparin/ clivarina | reviparin sodium |
| low molecular weight heparin/ lmwh | low molecular weight heparin |

Table 2S. The breakdown of the SMQ codes

| SMQ codes | SMQ name |
| --- | --- |
| 20000077 | congenital, familial, and genetic disorders |
| 20000186 | pregnancy, labor, and delivery complications, and risk factors |
| 20000190 | fetal disorders |
| 20000191 | neonatal disorders |
| 20000192 | termination of pregnancy and risk of abortion |
| 20000193 | normal pregnancy conditions and outcomes |

Table 3S. PTs of exposure-related pregnancy

| PT codes | PT name |
| --- | --- |
| 10071407 | maternal exposure during delivery |
| 10071409 | fetal exposure during delivery |
| 10071406 | maternal exposure before pregnancy |
| 10071408 | maternal exposure during pregnancy |
| 10071404 | fetal exposure during pregnancy |
| 10073513 | exposure during pregnancy |
| 10071415 | maternal exposure timing unspecified |
| 10071405 | fetal exposure timing unspecified |
| 10026923 | maternal drugs affecting fetus |
| 10064998 | drug exposure before pregnancy |

Table 4S. 2 × 2 contingency table of disproportionality method.

| Item | Target adverse events reported | Other adverse events reported | Total |
| --- | --- | --- | --- |
| Reports with the target drug | a | b | a+b |
| All other drugs | c | d | c+d |
| Total | a+c | b+d | a+b+c+d |

Table 5S. The principles of disproportionate measurement and the criteria for signal detection.

| Method | Calculation formula | ﻿Criteria |
| --- | --- | --- |
| ROR | $ROR=\frac{a / c}{b / d}$ | a ≥ 3  ROR ≥ 1  95%CI (lower limit) > 1 |
|  | $SE(lnROR)=\sqrt{\frac{1}{a}+\frac{1}{b}+\frac{1}{c}+\frac{1}{d}}$ |  |
|  | $95\%CI= e^{\ln\left( ROR \right)\pm1.96se}$ |  |
| PRR | $PRR=\frac{a / (a+b)}{c / (c+d)}$ | a ≥ 3  PRR ≥ 2  95%CI (lower limit) > 1 |
|  | $SE(lnPRR)=\sqrt{\frac{1}{a}-\frac{1}{a+b}+\frac{1}{c}-\frac{1}{c+d}}$ |  |
|  | $95\%CI= e^{\ln\left( PRR \right)\pm1.96se}$ |  |
| BCPNN | $IC=\log_{2}\frac{p(x, y)}{p\left( x \right)p(y)}= {log}_{2}\frac{a(a+b+c+d)}{(a+b)(a+c)}$ | IC025>0 |
|  | $E(IC)=\log_{2}\frac{(a+\gamma11)(a+b+c+d+\alpha)(a+b+c+d+\beta)}{\left( a+b+c+d+\gamma\right)(a+b+\alpha1)(a+c+\beta1)}$ |  |
|  | $V\left( \mathrm{IC} \right)=\frac{1}{{(ln2)}^{2}}[\frac{\left( a+b+c+d \right)-a+\gamma-\gamma11}{\left( a+\gamma11 \right)\left( 1+a+b+c+d+\gamma\right)}+\frac{\left( a+b+c+d \right)-\left( a+b \right)+a-\alpha1}{\left( a+b+\alpha1 \right)\left( 1+a+b+c+d+\alpha\right)}+\frac{\left( a+b+c+d+\alpha\right)-\left( a+c \right)+\beta-\beta1}{\left( a+b+\beta1 \right)\left( 1+a+b+c+d+\beta\right)}]$ |  |
|  | $\gamma=\gamma11\frac{(a+b+c+d+\alpha)(a+b+c+d+\beta)}{\left( a+b+\alpha1 \right)(a+c+\beta1)}$ |  |
|  | $IC-2SD=E\left( \mathrm{IC} \right)-2 \sqrt{V(IC)}$ |  |
| EBGM | $EBGM=\frac{a(a+b+c+d)}{\left( a+c \right)(a+b)}$ | EBGM05>2 |
|  | $SE(lnEBGM)=\sqrt{\frac{1}{a}+\frac{1}{b}+\frac{1}{c}+\frac{1}{d}}$ |  |
|  | $95\%CI= e^{\ln\left( EBGM \right)\pm1.96se}$ |  |

Abbreviations: BCPNN, bayesian confidence propagation neural network; CI, confidence interval; EBGM, empirical bayesian geometric mean; IC, information component; PRR, proportional reporting ratio; ROR, reporting odds ratio.
